# Surface-Attached Polyhistidine-Tag Proteins Characterized by FTIR Difference Spectroscopy

**DOI:** 10.1002/cphc.201200358

**Published:** 2012-06-15

**Authors:** Philipp Pinkerneil, Jörn Güldenhaupt, Klaus Gerwert, Carsten Kötting

**Affiliations:** a Lehrstuhl für Biophysik, Ruhr-Universität Bochum44780 Bochum (Germany)

**Keywords:** bilayers, difference spectroscopy, ir spectroscopy, proteins, reaction mechanisms

FTIR difference spectroscopy can reveal the molecular details of protein reactions.^[^1^–^6^]^ The absorption spectrum of a protein provides global information, for example, on protein secondary structure. In order to reveal molecular reaction mechanisms, difference spectroscopy has to be applied. It allows monitoring of functional groups that are involved in conformational changes, chemical reactions, H-bond changes, ligand binding or protein–protein interactions. In these difference spectra, the small signal of the reaction is selected out of the usually a factor of 10^3^ larger, but quiescent, background absorbance of the whole sample. Therefore identical conditions must be maintained between the measurements of two different states of the protein. The reaction can be triggered without moving the sample, by a laser flash. Although this is a powerful method for photoactive proteins like bacteriorhodopsin (bR)^[^7^]^ or the bacterial reaction center (RC),^[^8^]^ the function of most proteins cannot be triggered by light. One possible solution to overcome this problem is the use of caged compounds,^[^9^,^^10^^]^ which are photolabile precursers of active compounds. For example, caged GTP can be used for the investigation of GTPases.^[^11^]^

A more general method to perform difference spectroscopy is the use of immobilized proteins on attenuated total reflection (ATR) crystals.^[^6^,^^12^^,^^13^^]^ Difference spectra can then be obtained by simple buffer exchange, for example, with a ligand versus without. The group of Vogel modified Ge and ZnSe internal reflection elements (IREs) by gold or chemical vapor deposition of SiO_2_ and subsequently attached a linker molecule that reversibly binds peptides by a polyhistidine tag.^[^14^]^ During the last years SEIRA (surface-enhanced infrared absorption) spectroscopy of protein monolayers covalently immobilized on gold-coated silicon was established.^[^15^]^ Due to the gold layer this technique is superior for potential-induced difference spectroscopy. However, the strong near-field of the rough gold surface might hamper quantitative analysis, because the enhancement factor varies dramatically. We and others recently established ATR-FTIR difference spectroscopy of monolayers using a multiple reflection germanium ATR crystal as the internal reflection element (IRE).^[^13^,^^16^^,^^17^^]^ Due to the high refractive index, suitable chemical properties, and the good transmission in the mid-infrared region, germanium is probably the best IRE material in the mid-infrared region. Instead of the surface-enhanced effect in SEIRA, multiple reflection can be used and give similar signal to noise ratios.^[^17^]^ Further, germanium surfaces can easily be treated to become hydrophilic and the spreading of lipid vesicles results a stable lipid bilayer.^[^16^]^ Here, we report a method to immobilize proteins by means of a polyhistidine tag and lipids modified by nitrilotriacetic acid (NTA). Because polyhistidine tags are often used for protein purification,^[^18^]^ modified proteins are easy to access, which makes our method a universal technique for the investigation of almost any soluble protein.

**Figure 1:**
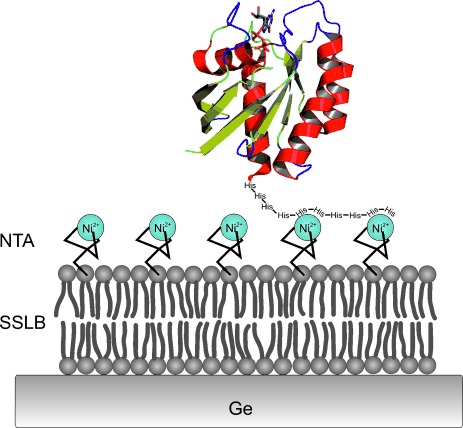
Schematic representation of our technique. A SSLB containing NTA-modified lipids is attached on top of a germanium ATR crystal by hydrophilic interactions. Any His-tag-modified protein can be immobilized and investigated by FTIR spectroscopy.

Our novel measurement platform is shown in Figure 1. First, a solid supported lipid bilayer (SSLB) is formed by vesicle spreading on top of an ATR crystal. We used small unilamellar vesicles consisting of a mixture of the natural 1-palmitoyl-2-oleoyl-sn-glycero-3-phosphocholine (POPC) and the head-group-modified lipid 1,2-di-(9Z-octadecenoyl)-sn-glycero-3-[(*N*-(5-amino-1-carboxypentyl)iminodiaceticacid)succinyl] (NTA-DOGS). The spreading of these vesicles resulted in a bilayer containing NTA groups, which enables protein immobilization via polyhistidine tags. Because the process of bilayer formation can be done within the spectrometer by using a flow-through system, we could easily monitor this process by the infrared absorptions of the lipids, and obtained the kinetics and the difference spectrum of the immobilization. From these data, surface concentration, lipid phase, and order parameters could be calculated, which then allowed the quantification of the bilayer quality. Larger vesicles did not spread completely and led to absorptions higher than expected for one bilayer (Supporting Information, Figure S1). On the other hand, bilayers prepared by small vesicles were stable and could be used for several days. We verified that the layer was complete (Supporting Information, Figure 2).

**Figure 2:**
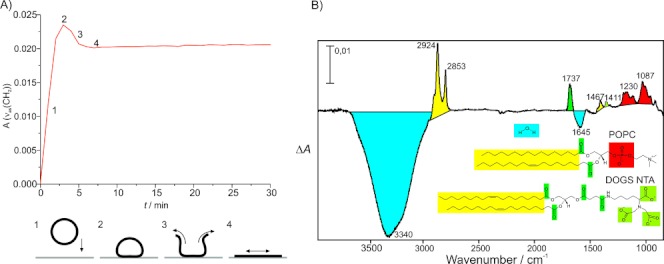
Formation of the lipid bilayer. A) Typical time course of vesicle spreading monitored by the CH_2_ stretching vibration of the lipid. B) Difference spectrum of the bilayer formation. Bands facing upwards are due to the bilayer, bands facing downwards are due to the displaced water.

Figure 2 A shows the time course of the lipid immobilization process with our optimized conditions. The adsorption of the vesicles was monitored by the absorbance of the asymmetric CH_2_ stretching vibration at 2924 cm^−1^. After 10 min, a stable absorption of 20 mOD was reached as expected for a single bilayer^[^17^]^ with a surface concentration similar to Langmuir–Blodgett films.^[^19^]^ The absorption difference spectrum of bilayer formation is defined as the difference between the infrared absorptions before and after this bilayer formation (Figure 2 B). Whereas the water displaced by the lipids produced negative peaks, all absorption bands of the lipids result in positive peaks in this difference spectrum. Besides the absorption of the asymmetric CH_2_ stretching vibration mentioned above, these positive peaks include the absorptions of the symmetric CH_2_ stretching vibration at 2853 cm^−1^, (asymmetric CH_3_ at 2958 cm^−1^ and symmetric at 2873 cm^−1^), of the carbonyl groups at 1737 cm^−1^, of the carboxylgroups of NTA at 1411 cm^−1^ and of the phosphates at 1230 cm^−1^ and 1087 cm^−1^. The band positions are typical for a lipid membrane in the liquid disordered phase. We further evaluated the quality of the bilayer by means of order parameters, which are a measure of the orientational distribution of the dipole moments of the absorption. These values (*S*=0.2 for the lipid alkane chain and *S*=−0.23 for the carbonyl absorption) are in line with literature values from NMR^[^20^]^ and IR.^[^17^]^

**Figure 3:**
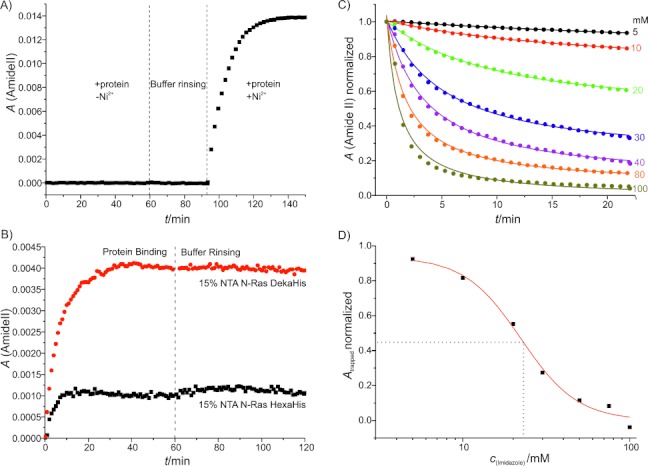
Protein immobilization. A) Typical time course of protein immobilization. We observed specific binding by the NTA groups, because no binding was observed without Ni^2+^. B) Immobilization of N-Ras 1-180 by the hexa- and deca-His tags. C) The protein can be removed by imidazole. D) IC50 of the displacement by imidazole.

In the next step, any polyhistidine-tagged protein can be immobilized. Again, this process can be directly monitored by the absorption bands of the adsorbed species. In this case, we used the amide II absorption of the protein (Figure 3 A). The protein only adsorbed if Ni^2+^ was present in the buffer, leading to a saturation of the surface within approximately 45 min, which indicated specific binding by the His tag. We optimized the conditions using N-Ras 1-180 with a C-terminal polyhistidine tag. In this case, the membrane anchoring via the polyhistidine tag mimicked the natural lipid anchor, which is also C-terminal and known to be less structured.^[^21^]^ We used either a hexa-His tag or a deca-His tag (Figure 3 B). The deca-His tag led to considerably more adsorbed protein, approximately four times that of the hexa-His tag under identical conditions with 15 % NTA groups. Using 25 % NTA-modified lipids and the deca-His tag, we obtained a signal of 14 mOD for the amide II band (Figure 3 A). This is close to the maximum absorption of 16 mOD obtained for lipid-anchored N-Ras, indicating a surface coverage of about 70 % for the Ras protein. The half-life time under our conditions was about 12 h.

The protein immobilization could be reversed by washing with imidazole (Figure 3 C), which provides additional proof for specific binding by the His tag. We used different concentrations of imidazole and found an IC_50_ of 22.5 mm for the deca-His tag and 25 mol % NTA-DOGS (Figure 3 D). This is between the values obtained for mono- and bis-NTA-modified polyethyleneglycol brushes.^[^22^]^ The reason for the stable immobilization is probably that the NTA groups are laterally mobile, which allows multivalent binding. This leads to a much stronger binding and prohibits the protein from coming off.^[^23^]^

**Figure 4:**
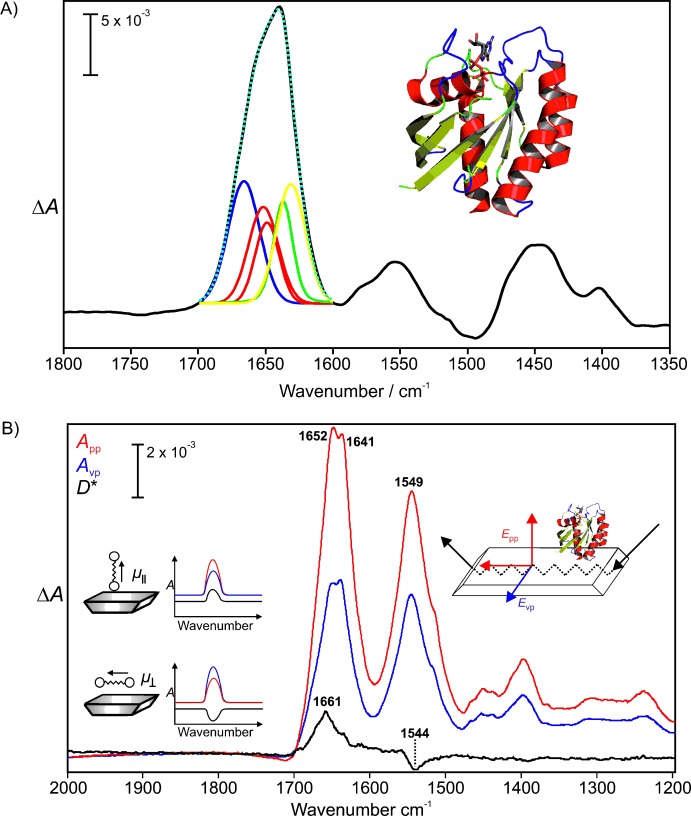
Applications of our technique. A) The FTIR spectrum of the protein reveals global features such as secondary structure by decomposition of the amide I band. B) By using polarized infrared light (*E*_pp_ and *E*_vp_), information on the orientation can be gained because absorptions with a transition dipole moment parallel to the perpendicular of the ATR crystal (*μ*_∥_) interact more strongly with *E*_pp_ and result a positive peak in the dichroic difference spectrum D*, whereas absorptions with a transition dipole moment parallel to the membrane (*μ*_⊥_) interact more strongly with *E*_vp_ and result a negative peak in D*. In D* of N-Ras1-166 a positive peak at 1661 cm^−1^ indicates that the majority of the transition dipole moments of the helical parts of the protein are perpendicular to the membrane. The transition dipole moment of helices is in the helix direction, resulting in an orientation as shown in Figure 1. A quantitative description for the calculation of orientations from D* is published elsewhere.^[^26^]^

Using this setup, several types of experiments are possible that can provide a variety of information. Again we exemplify these experiments using the GTPase Ras. By taking the spectrum before and after the protein immobilization process, the absorption spectrum of the protein was derived (Figure 4 A). From this, for example, the secondary structure of the protein can be obtained by decomposition of the amide I band.^[^16^]^ For these kinds of measurements, D_2_O should be used to prevent disturbance by the absorption signal of water. In our decomposition we found 33.4 % α-helix, 24.3 % β-sheet, 26.8 % β-turn and 15.4 % random coil. This agrees within the experimental error with both the X-ray structure^[^24^]^ and earlier FTIR measurement with lipid-anchored Ras.^[^16^]^ A similar amide I band was also obtained for K-Ras in solution.^[^25^]^ The slight increase in β-sheet and β-turn indicated that the peptide bonds of the additional ten NTA-bound histidine residues absorbed in this region.

Using polarized infrared light, dichroic measurements are possible. From this type of experiment, the orientation of functional groups relative to the membrane can be obtained. As shown in Figure 4 B (inset), an absorbing group with the transition dipole moment perpendicular to the membrane will absorb parallel polarized light more strongly (absorbance *A*_pp_) than vertically polarized light (absorbance *A*_vp_). This results in a positive peak in the dichroic difference spectrum D*, which is calculated by D*=*A*_pp_−*R*_iso_⋅*A*_vp_ (for details, see the Supporting Information).^[^17^]^ The positive peak in the amide I region, representing the helical content, shows that N-Ras1-166 was orientated as shown in Figure 4 B, with most helices perpendicular to the membrane. Thus, the orientation was similar to membrane-bound lipidated N-Ras.^[^26^]^ This indicates that in this case, the artificial binding not only immobilized the protein efficiently but even mimicked the natural anchor to a certain degree. Interestingly, N-Ras 1-180 does not show this orientation (Supporting Information, Figure S3). The longer linker enabled an isotropic distribution.

**Figure 5:**
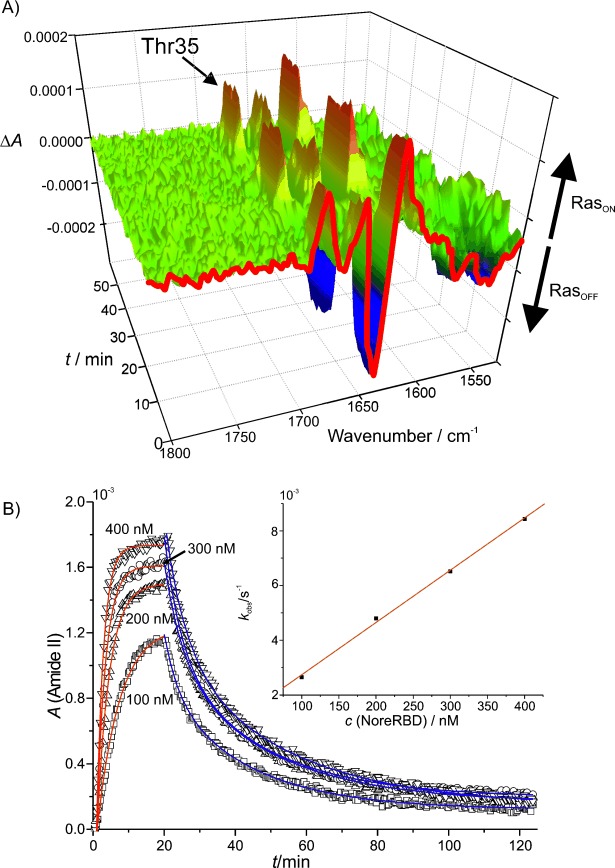
A) By simple buffer exchange, difference spectra are obtained. Here, the interaction of Ras⋅GDP with a small molecule (BeF_3_^−^) is shown. This molecule mimics the γ-phosphate and switches Ras into its “on” conformation as seen by the absorption of the marker band for the “on” state, the carbonyl vibration of Thr35.^[^28^]^ Repetitive changes between buffer with and without BeF_3_^−^ enable repetitive switching of the protein. B) Protein–protein interactions can be studied in a manner similar to surface plasmon resonance, but with the additional chemical information of the FTIR spectrum. Here, the association and the dissociation of NORE1A from Ras is shown. The raise can be fitted by a single exponential function. The plot of the time constant *k*_obs_ against the NORE1A concentration (inset) revealed the *K*_D_.

Furthermore, difference spectra can be obtained by utilizing the immobilization of the protein in a flow-through system (Figure 5 A). Thus, protein reactions could be induced in different ways, including changes in pH, addition or exchange of ligands, or small molecules. Here, we show the action of BeF_3_^−^ on Ras⋅GDP. BeF_3_^−^ mimics the γ-phosphate and led to a conformational change of the GTPase from its “off” into its “on” state.^[^27^]^ The observed difference spectrum is very similar to those obtained from Ras in solution and from lipidated membrane-bound Ras.^[^28^]^ For example, the Thr35 marker band in the “on” state at 1684 cm^−1^ (in D_2_O) is well-resolved. The reaction can be repeated many times to obtain signal-to-noise ratios from monolayers that are similar to the signal to noise from bulk experiments. Thus, reaction mechanisms can be investigated at the atomic level.

Another possibility is the use of the system in a surface-plasmon-resonance-type experiment, but gaining additional chemical information of infrared spectroscopy. Figure 5 B shows the association and dissociation of the effector protein NORE1A (199–358)^[^29^]^ to Ras using solutions with varying NORE1A concentrations. From these experiments, the *K*_D_ of the two proteins could be calculated. We obtained a value of 45 nm, which is in good agreement with the literature value of 80 nm.^[^29^]^ In addition, the infrared absorption spectrum of the second protein was obtained. Again, difference spectra of protein–protein interactions in various conditions, such as with and without small molecules, are possible.

In conclusion, we established a universal label-free method for the spectroscopic investigation of polyhistidine-tagged proteins. FTIR difference spectra can reveal reaction mechanisms of proteins at the atomic level. Usually, this method is limited to proteins with photoactivatable groups or redox active proteins. Our technique overcomes this limitation and allows FTIR difference spectroscopic investigations of any soluble or membrane attached protein by a simple buffer exchange. Further, information on secondary structure, molecular orientation, small-molecule or protein–protein interactions can be obtained.
