# Supplementary material for: Surface-Attached Polyhistidine-Tag Proteins Characterized by FTIR Difference Spectroscopy
Source: Chemphyschem. 2012 Jun 15;13(11):2649–53. doi: 10.1002/cphc.201200358 (PMC3531609; doi:10.1002/cphc.201200358)
Supplement: Supplementary file 1 [file cphc0013-2649-sd1.pdf]

CHEMPHYSCHEM

Supporting Information

© Copyright Wiley-VCH Verlag GmbH & Co. KGaA, 69451 Weinheim, 2012

Surface-Attached Polyhistidine-Tag Proteins Characterized by FTIR Difference Spectroscopy

Philipp Pinkerneil, Jörn Güldenhaupt, Klaus Gerwert,* and Carsten Kötting*[a]

cphc_201200358_sm_miscellaneous_information.pdf

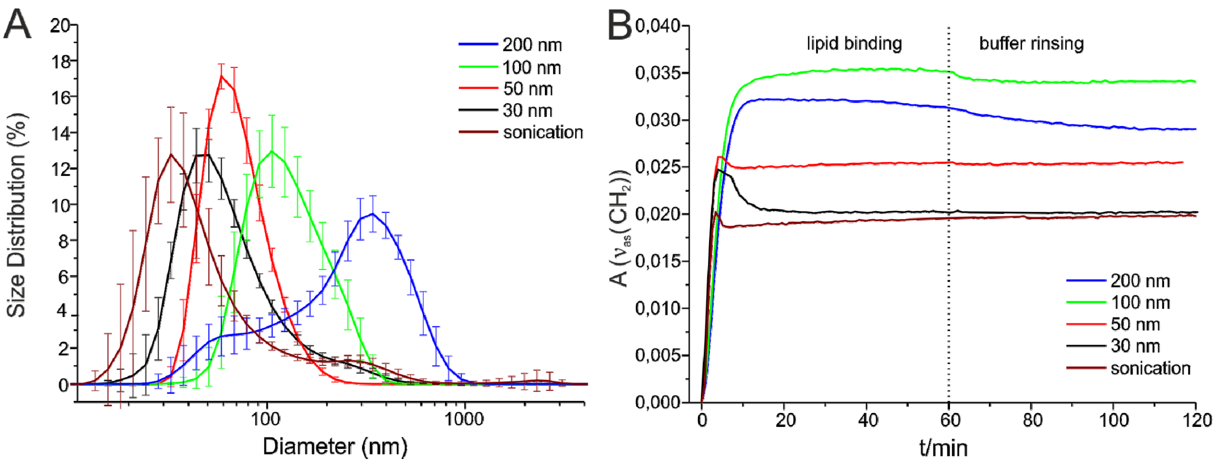

Supplemental Figure S1: Optimization of POPC vesicle spreading. (A) Vesicles of various sizes were produced by means of an extruder or by sonication. The sizes were determined by dynamic light scattering (Korgel et al., Biophys. J. 74, 3264 (1998)) using a Malvern HPPS (Malvern, Worcestershire, UK). (B) Time course of the vesicle spreading monitored by the CH_2 absorption of immobilized lipid at the ATR crystal. While small vesicles formed a single bilayer with the expected absorption of 20 mOD, larger vesicles gave higher absorptions, probably due to incomplete spreading. Vesicles of mixtures of POPC and NTA-DOGS spread more readily than pure POPC vesicles.

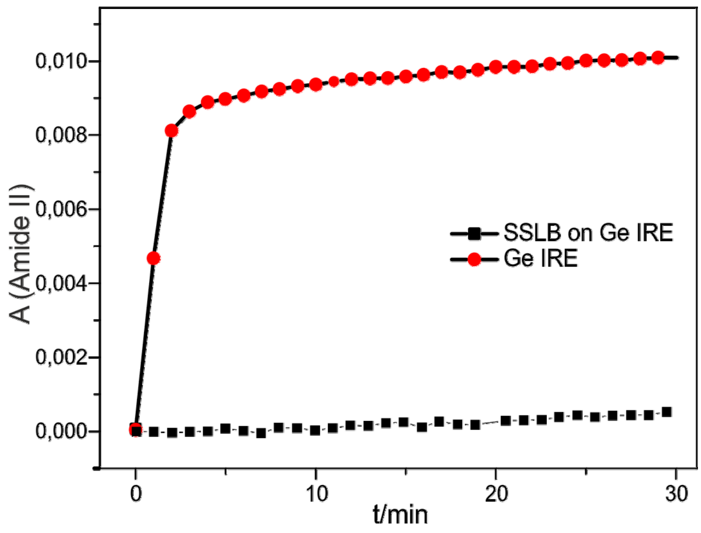

Supplemental Figure S2: Completeness verification of the SSLB. Bovine serum albumin (BSA) adsorbed readily at the hydrophilic germanium crystal (red). Thus BSA would also bind at any gaps within the lipid bilayer. In our control measurement (black) no further adsorption of BSA was detected, indicating a complete lipid bilayer. 0.8 µM BSA in buffer (20 mM Tris pH 7.4, 5 mM $MgCl_2$, 100 mM NaCl) were used for these experiments.

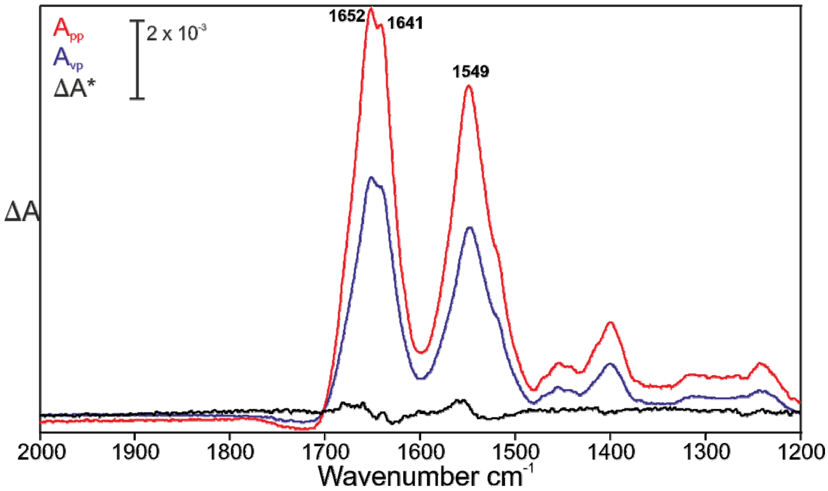

Supplemental Figure S3: Absorption spectra using polarized light (A_{pp} and A_{vp}) of N-Ras 1-180 and the calculated dichroic spectrum D*. No significant peak was observed in D* in the amide I region indicating an isotropic orientation of the protein.

Experimental Methods

N-Ras1–166His$_6$, N-Ras 1-166His$_{10}$, N-Ras 1-180His$_6$ and N-Ras 1-180His$_{10}$ were expressed as described for N-Ras without the His-tag.[1] The purification was carried out with immobilized metal ion affinity chromatography (IMAC) and size exclusion chromatography. For the experiments shown in Figure 4 N-Ras 1-166His$_{10}$ were used and for the experiments shown in Figure 5, N-Ras 1-180His$_{10}$ were used. NORE1A (199-358) was expressed and purified as a GST fusion protein according to the literature.[2] The GST-tag was removed by cleavage with thrombin.

The measurements were performed with a vertical ATR multireflection unit, (Specac, Orpington, UK) mounted in a Vertex 80V spectrometer (BrukerOptics, Ettlingen, Germany). The internal reflection element was a 52 mm · 20 mm · 2 mm trapezoidal germanium ATR plate with an aperture angle of 45°. Only one side of the IRE was used, which resulted in 13 active reflections. Measurements were done at 293 K with a resolution of 2 cm^{-1} and a scanner speed of 80 kHz using double sided forward backward data acquisition. Between 50 and 200 scans were averaged. Fourier transformation was done with a zero filling factor of 4, Mertz phase correction and Blackmann-Harris three-term apodization. Absorbances are calculated from the intensity I relative to the reference intensity I_0 (before the immobilization or reaction) according to $A = -\log(I/I_0)$. By means of a polarizing filter, we obtained absorption spectra for parallel polarized light (A_{pp}) and vertical polarized light (A_{vp}). The dichroic difference spectrum D* is calculated by $D^* = A_{pp} - R_{iso} \cdot A_{vp}$. R_{iso} corrects for the

different electric field strength for parallel and vertical polarized light. It was shown that under our experimental conditions $R_{iso} = 1.72$.[3]

POPC and NTA-DOGS were purchased from Avanti Polar Lipids (Alabaster, Alabama, USA). Vesicle formation bilayer preparation was done in the same manner as described for pure POPC.[3] The lipid mixture for the SSLB formation had a concentration of 410 μM (up to 25 mol% NTA-DOGS). The kinetics of the vesicle spreading was monitored by the asymmetric CH_2 stretching vibration between 2925 cm^{-1} and 2922 cm^{-1}.

Protein immobilization was achieved with a solution of 1 μM N-Ras in buffer (20 mM Tris pH 7.4, 5 mM $MgCl_2$, 100 mM NaCl, 0.1 mM $NiCl_2$, 1 mM TCEP, 0.1 mM GDP). The kinetics of the protein immobilization were monitored by the amide II mean absorption between 1550 cm^{-1} and 1545 cm^{-1}. Secondary structure analysis was done in D_2O with the same procedure and parameter set as used for lipidated Ras.[4] We used 4 mM BeF_2 and 16 mM NaF (1:6 molar ratio of beryllium to fluoride) for the generation of BeF_3^- to mimic the γ-phosphate in the protein activity tests. To determine the dissociation constant of the NORE1A·N-Ras complex, the apparent association rates (k_{obs}) were plotted against the concentration of NORE1A. The slope of the resulting regression line gives k_{on} and the intercept with the y-axis the k_{off}. The dissociation constant K_D is the quotient of these two rate constants.

[1] J. Tucker, G. Sczakiel, J. Feuerstein, J. John, R. S. Goody, A. Wittinghofer, *EMBO J.* **1986**, *5*, 1351.

[2] B. Stieglitz, C. Bee, D. Schwarz, O. Yildiz, A. Moshnikova, A. Khokhlatchev, C. Herrmann, *EMBO J.* **2008**, *27*, 1995.

[3] C. Kötting, J. Güldenhaupt, K. Gerwert, *Chem. Phys.* **2012**, *396*, 72.

[4] J. Güldenhaupt, Y. Adigüzel, J. Kuhlmann, H. Waldmann, C. Kötting, K. Gerwert, *Febs Journal* **2008**, *275*, 5910.
